# Infant and young child feeding in emergencies: Organisational policies and activities during the refugee crisis in Lebanon

**DOI:** 10.1111/mcn.12576

**Published:** 2018-01-08

**Authors:** Linda Shaker‐Berbari, Hala Ghattas, Andrew G. Symon, Annie S. Anderson

**Affiliations:** ^1^ School of Nursing and Health Sciences University of Dundee Dundee Scotland UK; ^2^ Faculty of Heath Sciences American University of Beirut Beirut Lebanon; ^3^ Centre for Public Health Nutrition Research University of Dundee Dundee UK

**Keywords:** breastfeeding, emergencies, health policy, infant and young child feeding, infant feeding, refugees

## Abstract

Appropriate infant and young child feeding (IYCF) is key to reducing mortality amongst children aged under 2. Facilitating adherence to recommended IYCF practices during emergencies includes having relevant policies to support breastfeeding and complementary feeding as well as regulating the distribution of breast milk substitutes. In the current crisis, more than 1.2 million Syrian refugees are in Lebanon and it is timely to examine organisational IYCF policies and programmes. One hundred and thirty‐five non‐governmental organisations providing humanitarian aid in Lebanon were invited to participate in an online survey about organisational policies and programmatic activities on IYCF. Responses were obtained from 54 organisations: 29 International Non‐Governmental Organisations (INGOs) and 25 Local Non‐Governmental Organisations (LNGOs). In total, 8 (15%) reported having a written policy on IYCF, but only 1 policy (in draft format) was available for inspection. Twelve (8 INGOs and 4 LNGOs) indicated endorsing an external IYCF policy, but only 6 listed a valid policy. Four organisations (3 INGOs and 1 LNGO) had programme objectives that indicate protection, promotion, and support of IYCF. Three LNGOs reported receiving infant formula donations and 5 organisations (2 INGOs and 3 LNGOs) indicated distributing infant formula; 2 (1 INGO and 1 LNGO) did so in accordance with international and national policies. Few organisations violated IYCF guidance but organisational policies and activities on IYCF are not well established. In order to improve response in the current refugee crisis in Lebanon, there is a need to ensure policies are in place and implemented so that interventions support, promote, and protect IYCF.

**Key messages**

- During the current Syria refugee crisis, few of the surveyed local and international organisations providing humanitarian aid in Lebanon have written infant and young child feeding (IYCF) policies or endorse a valid external IYCF policy.
- Five organisations distributed infant formula; one did so in accordance with international and national policies.
- IYCF policies and activities are not well established and there is a need to ensure that policies are put in place and implemented so that IYCF interventions support, promote, and protect IYCF.

## 1 INTRODUCTION

Appropriate infant and young child feeding (IYCF) is essential for adequate growth, development, and survival. The World Health Organisation (WHO) recommends exclusive breastfeeding for the first six months of age, thereafter introducing adequate complementary food, and ensuring breastfeeding is continued up to the age of two and even beyond (WHO & UNICEF, 2003). Worldwide, about 45% of child mortality has been reported to be related to malnutrition, with more than 800,000 deaths of infants and young children attributable to poor IYCF practices (Black et al., 2013).

Facilitating adherence to recommended IYFC practices becomes vital in emergency situations (e.g., civil unrest and natural disaster) where access to health care, clean water, and adequate nutrition can be compromised (WHO & UNICEF, 2003). The importance of breastfeeding becomes even greater in such contexts. For example, following the South East Asian tsunami in 2004, rates of diarrhoea were 3 times higher amongst children who were artificially fed compared to those who were breastfed (Adhisivam, Srinivasan, Soudarssanane, Deepak Amalnath, & Nirmal Kumar, 2006). During the Bosnian war in the 1990s, nonbreastfed infants under 4 months of age were reportedly more likely to become malnourished than their breastfed counterparts (Andersson, Paredes‐Solís, Legorreta‐Soberanis, Cockcroft, & Sherr, 2010). In war‐torn Iraq, Al‐Sharbatti and AlJumaa (2012) reported that formula‐fed infants had a 2.7‐fold increased risk for acute respiratory infections compared to breastfed infants.

Despite the established value of supporting infant and young child feeding in emergencies (IYCF‐E), IYCF practices continue to be undermined by inappropriate distribution of infant formula, milk, or milk products as well as the lack of support for breastfeeding mothers (Carothers & Gribble, 2014; Gribble, McGrath, MacLaine, & Lhotska, 2011). Recognising the importance of supporting IYCF in emergencies has led to a number of strategies, frameworks, policies, and guidance which set out responsibilities for actors during emergencies related to IYCF. Resolution 63.23 published in 2010 by the World Health Assembly urges member states to follow the Operational Guidance (OG) for Emergency Relief Staff and Programme Managers on IYCF‐E (IFE Core Group, 2007). The guidance includes the protection, promotion, and support for breastfeeding and “the need to minimise the risk of artificial feeding by ensuring that required breastmilk substitutes are purchased, distributed and used according to strict criteria.” As per the OG, infant formula should only be provided to mothers or caregivers of infants who need them and donations of breast milk substitutes including bottles and teats should not be accepted (IFE Core Group, 2007). Although there are several examples of policy guidance on supporting IYCF‐E, gaps in implementation are known to exist (Gupta et al., 2012). For example, during the Balkan crisis in the 1990s, it was estimated that around 1.4 metric tons of baby food were donated in the first weeks of the emergency in violation of IYCF‐E policies (Borrel et al., 2001).

In the context of the Syrian crisis, Lebanon has been hosting around 1.2 million refugees from Syria (UNHCR, 2016). Prior to this refugee influx, Lebanese authorities had enacted the International Code of Marketing of Breast milk Substitutes (the Code) which also applies in emergencies (WHO, 1981). Law 47 in Lebanon, issued in 2008 and titled “Organizing the Marketing of Infant and Young Child Feeding Products and Tools” is the main official document that includes relevant guidance on IYCF. It emphasises the three pillars of appropriate IYCF—to protect, promote, and support breastfeeding—and stresses the importance of ensuring safe use of complementary food and products (Republic of Lebanon Parliament, 2008). However, the Law does not refer specifically to emergencies nor does it emphasise the importance of applying the law in emergency situations. In response to the increasing numbers of refugees crossing from Syria into Lebanon in 2012, a joint statement was issued by the international humanitarian community (Non‐Governmental Organisations and UN agencies) with the Ministries of Public Health and Social Affairs (Ministry of Public Health [MoPH] et al., 2012). The statement was also considered a policy document serving as an addendum to the Lebanese Law. However, no publication of the joint statement appeared in the official gazette to render it official.

Save the Children UK's report on IYCF in Lebanon during the 2006 Israel–Lebanon conflict noted that a lack of awareness of the OG and the Code resulted in Code violations (i.e., untargeted distribution of infant formula to affected displaced populations (Maclaine, 2006). The report also highlighted a lack of commitment from International Non‐Governmental Organisations (INGOs) and UN agencies to the Code and OG and a failure to support mothers to continue breastfeeding during and after the conflict. The report asserted that infant feeding was not a priority for lead agencies. Recently, Akik, Ghattas, Filteau, and Knai (2017) identified barriers to breastfeeding in Lebanon including gaps in enforcement of Law 47. They reported a gap between policy endorsement and translation on the ground where the existing law was not properly disseminated nor institutionalised. On the other hand, Akik et al. (2017) also highlighted the role of international organisations in supporting IYCF through the MoPH.

Currently, a large number of humanitarian agencies are engaged in providing support to Syrian refugees in the four regions of Lebanon (North, Bekaa, South and Beirut and Mount Lebanon). With already existing lack of adherence to recommended feeding practices (WFP et al., 2016), it is timely to examine the extent to which organisations abide by policies related to IYCF in emergencies to ensure that IYCF standards are upheld.

The current paper examines policies and programmatic activities of NGOs active in the humanitarian field in Lebanon in response to the Syrian refugee crisis.

## 2 PARTICIPANTS AND METHODS

An online survey was designed and tested for content and face validity in English and Arabic. The following survey modules were included: basic information of organisation (area of operation and type of activities), IYCF policies (organisational or international), IYCF programmatic activities, and donations and distribution of infant formula and milk.

At the time of recruitment, there were 464 international and national NGOs in Lebanon registered within the Ministry of Interior. According to the UNHCR online portal for Lebanon, around 90 organisations were active in the provision of humanitarian assistance to Syrian refugees of which around 45 were active in health, food security, or nutrition (UNHCR, 2016); the precise number fluctuates from one period to another. This study included all these agencies in addition to local organisations that were identified through the Lebanese NGO portal (http://daleel-madani.org). UN and government agencies were not included. One hundred thirty‐five identified organisations registered as working in the following fields were invited to participate in the survey: (a) Food and Nutrition, (b) Relief Services, (c) Refugees, (d) Labour and Livelihood, (e) Health and Family. An email was sent to the head of each organisation inviting the organisation to be involved in the study and requesting completion of a survey (online in English or by email in Arabic) by either the relief coordinator, programme coordinator, programmes manager, or country representative. Recipients were advised that the respondent should be directly involved and in charge of relief activities in the organisation. The email included a participant information sheet with details about the research objectives and a confidentiality note. Given that the responses were requested electronically, the sheet clarified that a completed questionnaire was considered as consent to participate under the terms of the participant information sheet. The questionnaire also had a question confirming that the participant agreed to proceed with filling the questionnaire. Where necessary, three‐reminder emails were sent to encourage participation. Survey responses were entered into Microsoft Excel. Each organisation was given an identification number (INGO# or LNGO# depending on whether they are international or local) in order to ensure confidentiality. Basic descriptive analysis was conducted and open ended questions related to organisational programme objectives were coded and entered. Objectives were coded on the basis of the three pillars of IYCF as mentioned in the Global Strategy for Infant and Young Child Feeding (WHO & UNICEF, 2003). Accordingly, programme objectives were categorised as either belonging to “IYCF promotion,” “IYCF support,” or “IYCF protection” depending on the nature of the activity described. The research was approved by the University of Dundee Research Ethics Committee.

## 3 RESULTS

Of the 135 NGOs that were sent the questionnaire, 11 (8%) failed to be delivered (incorrect email addresses). Therefore, it is assumed that 124 NGOs received the correspondence. Sixty‐four NGOs (47%) responded, 54 (44%) completed the questionnaire, 9 (7%) responded that the questionnaire did not apply to their work, and one replied to say that they declined to participate. Fifty‐three organisations answered online in English and one answered the Arabic language questionnaire by email.

**Figure 1:**
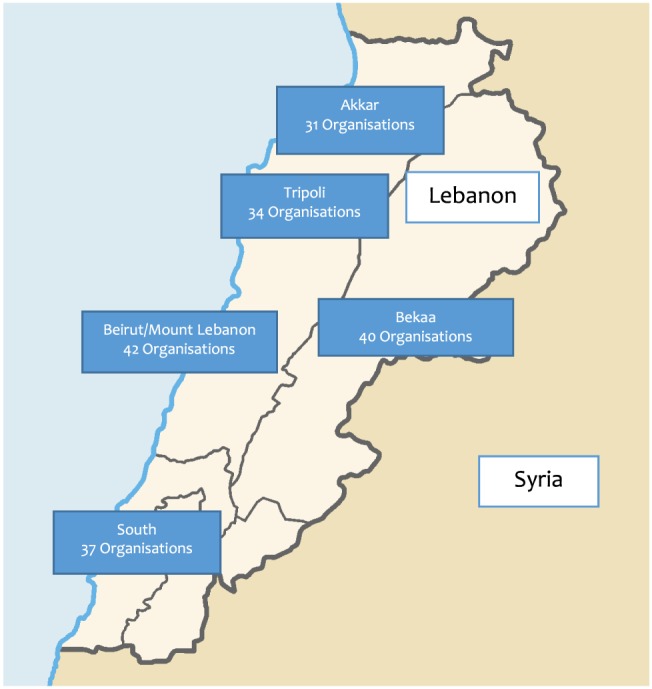
Map of Lebanon and surveyed Non‐Governmental Organisations area of operation

Of the 54 organisations, 29 (54%) were INGOs (response rate 57%) and 25 (46%) Local Non‐Governmental Organisations (LNGO; response rate 34%). The 54 organisations were divided based on their area of operations: 28 reported working at the national level, 14 reported working in more than one geographic region, and 12 reported working only in one geographic region. Figure 1 shows the number of NGOs that work in each of the regions.

### 3.1 IYCF policies and programmatic activities

The organisations' basic activities varied, extending from provision of nonfood items (kitchen utensils, beddings, diapers, etc.) to school education and health care (including nutrition and food security; Table 1). Twenty‐nine (19 INGOs and 10 LNGOs) out of the 54 organisations that responded to the questionnaire reported implementing programmes related to health (general health services), reproductive health (prenatal and postnatal care), or nutrition of which 18 (12 INGOs and six LNGOs) reported targeting pregnant and lactating women (PLWs) and infants and young children (IYC). Of those targeting PLWs and IYC, 14 (INGOs and LNGOs) reported having a system or programme for the promotion, support, and protection of IYCF. However, based on reported objectives, only four organisations (three INGOs and one LNGO) had objectives that indicated activities that can be categorised under all three components: promotion, protection, and support of IYCF. The rest were engaged in some form of IYCF‐related activity such as health education.

**Table 1:** Type of activity*

Type of activity organisation is engaged in		
	*N*	%
Distribution of nonfood items	32	59
Health–general	21	39
WASH^1^	18	33
Education	18	33
Other	17	31
Health–reproductive health	17	31
Distribution of food items	16	30
Food security and livelihood	12	22
Health–nutrition	11	20
Protection	3	6

*Note*.

* The types of activities are based on the sectors listed under the Sphere project.^2^

Organisations that engaged in health (general health services), nutrition, food security, distribution of food and nonfood items, water, sanitation, and hygiene, or other related activities were categorised as benefiting from having an IYCF policy. However, of the 44 organisations engaged in these activities, only eight (five INGOs and three LNGOs) responded having a written policy on IYCF within their own organisation. The rest did not have an internal policy (20 INGOs and 21 LNGOs) or did not know (four INGOs and one LNGO).

Of the eight organisations that said they had an IYCF policy, four were willing to share this and three were not. The eighth (LNGO‐44) replied that the question was not applicable. The four organisations which indicated willingness to share their policy were sent an email requesting a copy. One (INGO‐40) sent the policy, indicating it was still a draft; INGO‐45 confirmed having a policy but needed to double check preparedness to share this, whereas INGO‐29 indicated that its policy was still in preparation and could not be shared. The fourth (LNGO‐47) did not respond. In summary, only one policy was actually available for inspection, and it was in draft format.

In terms of endorsing an external IYCF policy, 12 (eight INGOs and four LNGOs) out of the 44 organisations answered “yes.” Five of these (three INGOs and two LNGOs) were amongst the eight that indicated having an internal policy. When asked to indicate the name of the endorsed policy, 13 organisations responded (seven INGOs and six LNGOs), two of which had not previously indicated that they did endorse an external policy. One organisation that had indicated endorsing an external policy did not answer the question related to naming the policy.

Of the 13 organisations that answered this question, some mentioned more than one policy. Six mentioned existing policies and guidance, the rest had incomplete answers.^3^

In summary, the policies and guidance reported were the OG, the SPHERE standards, the Code, and the Inter‐Agency Standing Committee Core Commitments for Children in Emergencies; Statement 47 of the Lebanese law and WHA 34.22. Therefore, only 11 (20%) organisations could be confirmed as having their own policy (*n =* 5), endorsing a valid external policy on IYCF (*n =* 3) or both (*n =* 3).

### 3.2 Donations and distribution of infant formula and milk

Three LNGOs working in Bekaa (LNGO‐01 and LNGO‐47) and Beirut and Mount Lebanon (LNGO‐35) reported receiving donations of infant formula from an external source whereas two LNGOs indicated they did not know whether their organisation ever received such a donation. None of the organisations reported discarding the donation and none reported on the quantity received. All three reported distribution of the donated formula “to infants within the general distribution” (LNGO‐35, LNGO‐01, and LNGO‐47) and “to families of nonbreastfed infants” (LNGO‐01 and LNGO‐47). LNGO‐47 added also “donating to MoPH.”

Regardless of whether organisations received donations, five indicated distributing infant formula (three of which were the same NGOs as those receiving donations). One LNGO answered they did not know whether their organisation distributed any infant formula to families. The two organisations that did not receive infant formula distributed to selected mothers and infants; the rest used mass distribution. Tables 2 and 3 show a summary of responses received from the five organisations that indicated distributing infant formula.

**Table 2:** Programmatic activities and policy status of organisations engaged in provision of infant formula

	LNGO01	INGO33	LNGO35	INGO40	LNGO47
Geographical coverage	One region	National	Two regions	National	One region
Programmatic activities					
Distribution of food items	Y	‐	Y	Y	Y
Distribution of nonfood items	Y	‐	Y	Y	‐
Reproductive health	Y	Y	‐	Y	‐
Health	Y	Y	‐	Y	‐
Food security and livelihood	Y	‐	‐	Y	Y
Education	Y	‐	Y	Y	Y
Nutrition	‐	‐	‐	Y	‐
WASH	‐	‐	‐	Y	Y
Organisation targets PLWs and IYC	Y	Y	Y	Y	Y
Activities targeting PLWs and IYC					
Distribution of relief items	Y	‐	‐	Y	Y
Support for primary health	Y	Y	‐	Y	Y
Health awareness	Y	Y	‐	Y	Y
Capacity building	‐	‐	Y	Y	Y
Organisation reports having a system for promotion, support and protection of IYCF	DNK	N^Not aligned^	N^Not aligned^	Y^Aligned^	N^Not aligned^
Evaluation of programme objectives					
Programme objectives include IYCF promotion	N^Not aligned^	N^Not aligned^	N^Not aligned^	Y^Aligned^	N^Not aligned^
Programme objectives include IYCF support	N^Not aligned^	N^Not aligned^	N^Not aligned^	Y^Aligned^	N^Not aligned^
Programme objectives include IYCF protection	N^Not aligned^	N^Not aligned^	N^Not aligned^	Y^Aligned^	N^Not aligned^
Policy status					
Organisation has a written IYCF policy?	N^Not aligned^	N^Not aligned^	N^Not aligned^	Y^Aligned^	Y^Aligned^
IYCF policy shared?	N/A	N/A	N/A	Y^Aligned^	N
Organisation endorses an external IYCF policy?	N^Not aligned^	N^Not aligned^	N^Not aligned^	Y^Aligned^	Y^Aligned^
Organisation endorses a valid external policy?	N/A	N/A	N/A	Y^Aligned^	N^Not aligned^

*Note*. Y = yes; Aligned = Aligned with IYCF‐E guidance; N = no; Not aligned = not aligned with IYCF‐E guidance; N/A = not applicable; DNK = does not know; NGO = Non‐Governmental Organisations; LNGO = Local Non‐Governmental Organisations; WASH = water, sanitation, and hygiene; PLWs = pregnant and lactating women; IYC = infants and young children; IYCF = infant and young child feeding.

**Table 3:** Mode of provision of infant formula amongst five organisations engaged in provision of infant formula

	LNGO01	INGO33	LNGO35	LNGO40	LNGO47
Handling of breast milk substitutes					
Receipt of donation					
Organisation received donation of infant formula?	Y^Not aligned^	N^Aligned^	Y^Not aligned^	N^Aligned^	Y^Not aligned^
Quantity of donation	N/I	N/A	N/I	N/A	N/I
Mode of distribution of donation					
Mass/blanket distribution	Y^Not aligned^	N/A	Y^Not aligned^	N/A	Y^Not aligned^
Targeted distribution	Y^Aligned^	N/A	N	N/A	Y^Aligned^ *
Distribution of infant formula					
Organisation engaged in distribution of infant formula?	Y	Y	Y	Y	Y
Organisation is currently distributing infant formula?	Y	Y	N	Y	N
Duration expected to continue distribution?	N/I	4 years	N/A	Depends on funding	N/A
Mode of distribution of infant formula					
Mass/blanket distribution	Y^Not aligned^	Y^Not aligned^	N^Aligned^	N^Aligned^	Y^Not aligned^
Targeted distribution	N	N	Y^Aligned^	Y^Aligned^	N
#of families/infants benefiting from infant formula	3500	2000	675	60	N/A
# of times in the last 6 months	N/I	2×	1×	N/I	N/I
Organisation assessed infants prior to provision of infant formula?	Y^Aligned^	N^Not aligned^	N^Not aligned^	Y^Aligned^	Y^Aligned^
Infant formula was purchased?	N^Not aligned^	Y^Aligned^	Y^Aligned^	Y^Aligned^	N^Not aligned^
Infant formula was donated?	Y^Not aligned^	N^Aligned^	Y^Not aligned^	N^Aligned^	Y^Not aligned^
# of cans distributed?	N/I	N/I	N/I	1600	N/I
Infant formula labelled in Arabic?	Y^Aligned^	Y^Aligned^	Y^Aligned^	Y^Aligned^	N^Not aligned^
Brand name of infant formula displayed?	Y^Not aligned^	Y^Not aligned^	Y^Not aligned^	N^Aligned^	N^Aligned^
Distribution of milk powder					
Organisation distributed powdered milk (cow milk)	Y^Not aligned^	N^Aligned^	Y^Not aligned^	N^Aligned^	N^Aligned^
# of families targeted?	1100	N/A	1320	N/A	N/A
# of distributions in the last 6 months?	78×	N/A	2×	N/A	N/A
Violations to IYCF policy					
Organisation in violation of the code/law 47/2008/joint statement based on above activities?	Y	Y	Y	N	Y

*Note*. Y = yes; Aligned = Aligned with IYCF‐E guidance; N = no; Not aligned = not aligned with IYCF‐E guidance; N/A = not applicable; NI = not indicated.

* This organisation also indicated donating to MoPH.

The table describes the nature of the activities that these five organisations were engaged in as well as the extent to which the distribution of infant formula was in line with IYCF‐E policies and guidance. In summary, only one organisation had a programme with objectives encompassing the three pillars of promotion, support, and protection. That same organisation was the only one distributing infant formula in compliance with IYCF‐E policies and guidance.

## 4 DISCUSSION

This is the first study to examine IYCF policies and programmatic activities within local and international organisations active in Lebanon during the current response to the Syrian crisis. Findings show that despite the diversity of interventions, IYCF programming and policies are still limited to very few organisations.

Despite a considerable number of organisations working in health and targeting PLWs and IYC, few organisations had a comprehensive IYCF‐E programme. Four organisations involved in providing health care services, prenatal or postnatal care or nutrition to PLWs and IYC did not have any component in their programme addressing IYCF‐E. This reflects a lack of attention to IYCF‐E and an assumption on the part of organisations that infant nutrition and health can be supported without addressing IYCF. IYCF programmes have been reported to be most effective when integrated alongside other interventions such as maternal and child health (Save the Children & UNHCR, 2015; UNICEF, 2011). A multi‐sectorial approach that integrates nutrition with other sectors such as health creates efficiencies and is currently promoted by various movements in the field of nutrition (Duggan, 2014; GNC, 2014; Save the Children & UNHCR, 2015). It was apparent that the organisations' understanding of promoting, supporting, and protecting IYCF was poor. Although many indicated that they had such a system, analysis of their programme objectives showed that their activities did not cover all three pillars. Most of the activities focused on promotion such as provision of awareness and very few included support such as counselling or protection such as advocating for or monitoring the enforcement of IYCF policies. The implementation of IYCF programmes during emergencies requires human resources and sets of skills and competencies to ensure provision of counselling and support (Meeker et al., 2014). It may be that such capacities and resources may not have been available within organisations. Alternatively, organisations may not have prioritised such an intervention despite the evidenced contribution of IYCF interventions to decreasing child mortality and morbidity (Victora et al., 2016).

Very few organisations had an internal written IYCF policy and only one was able to provide an example. The first recommendation in the OG is for organisations to have an established internal policy on IYCF as a preparedness measure (IFE Core Group, 2007). Very few organisations endorsed a valid international or local policy. Lebanon has endorsed the Code through law 47/2008 which regulates the marketing of breast milk substitutes (Republic of Lebanon Parliament, 2008; World Breastfeeding Trend Initiative [WBTI], 2015); however, only one NGO actually cited this law. The rest named international guidance with the Code being the most commonly cited. In addition, a joint statement on IYCF was issued in 2012 that includes guidance on IYCF in the current crisis (MoPH et al., 2012). It appears that two organisations referred to the joint statement, even though one was able to give the correct name for it. The fact that only one organisation representative was aware of the local Lebanese law related to the marketing of breast milk substitute is an indication that policies did not trickle down to lower levels of implementation. Maclaine and Corbett (2006) also reported that INGO staff in Lebanon, questioned about their organisation's policy on infant feeding in emergencies, did not report awareness of the policy or its details. This lack of awareness about existing policies presents a risk for poor implementation of IYCF policies and suggests the need to further increase awareness of policies including the law amongst organisations.

Few organisations reported receiving donations of infant formula all of which were LNGOs working in well‐defined areas. Although in this study, NGOs did not specify the amounts of donations received, the number of families receiving donated infant formula was relatively small. This might indicate that the quantity received was low in comparison to other emergencies. For example, in the Balkans, Borrel et al. (2001) documented that in the early days around 3,500 metric tons of humanitarian aid was donated of which an estimated 40% was baby food. More recently in Syria, one of the largest humanitarian organisations in Damascus received a donation of 40 metric tons including baby milk (Syrian Arab Red Crescent, 2015).

Most organisations that dealt with infant formula, did so in violation of the Code, the OG, and Law 47/2008. Article 6.2 of the OG, the Joint Statement and Law 47/2008 all clarify that there should be clear criteria for targeting and use of breast milk substitutes. Of the five organisations that handled breast milk substitutes, only one was consistent in targeting selected mothers and infants (as opposed to untargeted blanket donations) and only 60 infants were targeted. For the other four and as indicated in Table 3, infant formula was distributed to up to 3,500 families by one organisation or more than 6,000 families by organisations collectively. Four organisations distributed infant formula that is branded and labelled in Arabic and one violated the guidance. It should be noted that the guidance (OG, Code) was violated by distributing infant formula labelled in a language different from that used by recipients. It is therefore evident that even with national policies and statements in place, these were not well implemented or reflected in the work being undertaken. This is consistent with the findings of Borrel et al.'s (2001) Balkans study which identified a lack of translation from policy to practice due in part to weak institutionalisation of policies, the absence of monitoring systems, and inadequate coordination mechanisms.

In Lebanon in 2006, Maclaine (2006) reported similar findings indicating violations of the Code and the IYCF OG. In that study, infant formula was distributed within baby kits by at least one INGO and three LNGOs, and around 1,500 baby kits were distributed (Maclaine, 2006). The numbers in Table 3 suggest that the magnitude of violations is greater than in 2006. However, it is also worth noting that the current refugee crisis is much greater than the 2006 emergency in Lebanon, with many more people affected (UNHCR, 2016).

Compared to other crises, the violations may be considered limited where thousands have been reported to receive untargeted infant formula; in the case of Lebanon, this constitutes less than 1% of the affected population (UNHCR, 2016). In Jakarta for example and after the earthquake, Hipgrave, Assefa, Winoto, and Sukotjo (2012) showed that 80% of affected families received infant formula. Similarly, in Iraq during the 2003 war, breast milk substitutes were included in the food basket, which affected mothers' decision to breastfeed (International Study Team, 2003). It is worth noting, however, that design of the studies was different in terms of reporting on violations because the current study collected information directly from organisations through an online survey, whereas most other studies looked at violations through surveys with mothers and health care providers (McInnes, Wright, Haq, & McGranachan, 2007). For example, Aguayo, Ross, Kanon, and Ouedraogo (2003) reported on violations in West Africa through a survey with health facilities and distribution points. Similarly, Maclaine (2006) communicated with mothers, health care providers, and NGO workers on distribution of infant formula to affected populations. Sometimes direct observations are also adopted such as in China where Liu, Dai, Xie, and Chen (2014) included observation as part of their data collection methods. Therefore, one might argue that violations may be under‐reported by organisations, whereas observations and reporting from beneficiaries allow for a more valid data collection method. Our study is part of a wider research project that includes information from mothers and primary health services and such data will shed further light on additional violations or confirm current findings.

At the same time, the limited support for nonbreastfed infants is important to address. Our findings show that only one NGO targeted families with artificial feeding support in line with recommendations (60 families were involved). The World Food Program (WFP, UNHCR, and UNICEF, 2016) reported that 45% of infants are exclusively breastfed amongst the Syrian refugee population in Lebanon, highlighting a high percentage of nonbreastfed infants in need of support. Previously, Dolan, McGrath, and Shoham (2014) reported on IYCF interventions during the current response in Lebanon and other countries and claimed that interventions in nutrition in Lebanon “missed the point.” Dolan et al. (2014) noted that few interventions focused on breastfeeding but also that the needs of nonbreastfed infants were not met through targeted artificial feeding support.

Overall, the limited IYCF activities and lack of policies indicate a need to upscale action to respond to the needs of infants and young children. Despite the large pool of organisations that may be expected to work on IYCF, very few had programmes established. The concept of championing has been discussed in different situations where it has been linked to the success of national initiatives and has been included in recommendations for advocating nutritional and IYCF initiatives (Ashworth & Jackson, 2011; Kathumba, 2012; Sumner, Lindstrom, & Haddad, 2008). It could be that the presence of such a champion in Lebanon would contribute to improving policies and programmatic activities on IYCF. Our findings show that there is potentially at least one organisation that is dedicated to upholding IYCF and there is potential that such organisations might play a role in improving IYCF interventions.

Although organisations that responded had initiatives throughout the country, still, it should be pointed out that only half the contacted organisations responded. This may be due to the heavy workload that organisations are engaged in during the refugee response. International organisations were more likely to respond to the survey, which may reflect greater experience with research participation and/or greater overall capacity.

For a few organisations, there was some lack of clarity about whether they had a written IYCF policy or not. Although it was requested that the survey was completed by appropriate staff, it is possible that respondents may have not been familiar with existing organisational IYCF policies or terminologies including what a policy is. Within questions related to endorsing or having a policy, there were apparent contradictions in the answers between questions that were purely objective (yes and no answers) and questions that required an elaboration (such as naming or sharing the policy). This is also valid for questions related to programming on IYCF.

## 5 CONCLUSION

Our findings show that despite the large number of organisations targeting infants and young children, IYCF‐E is not being given priority within organisational programming. Very few organisations had established policies related to IYCF in the current refugee crisis in Lebanon. IYCF interventions were limited to promotion of breastfeeding but not support or protection for breastfeeding mothers. Violations to national and international guidance occurred mainly in local organisations and IYCF was rarely integrated within programmes despite the fact that many organisations target PLWs and infants and young children.

In order to improve response during the current refugee crisis in Lebanon, and given the importance of ensuring adherence to recommended IYCF practices during emergencies, there is a need to ensure that policies are implemented within organisations that they guide everyday practice and that interventions support, promote, and protect IYCF.

## CONFLICTS OF INTEREST

The authors declare that they have no conflicts of interest.

## CONTRIBUTIONS

LS study concept design, field work design, data collection, analysis and interpretation, and MS drafting. AGS and ASA advisors on study design, analysis and interpretation, and MS editing. HG local advisor on study design and data collection and interpretation.

## Footnotes

1 Water Sanitation and Hygiene Promotion

2 Sphere Project. Humanitarian Charter and Minimum Standard in Humanitarian Response. 2011.

3 An incomplete answer means either no response or a response that did not specify a policy. For example, answers such as “the latest one” and “protection” were considered incomplete answers.
